# Habitat use by semi-feral Konik horses on wetlands—three-year GPS study

**DOI:** 10.1007/s10661-023-11605-y

**Published:** 2023-08-11

**Authors:** Chodkiewicz Anna, Prończuk Martyna, Studnicki Marcin, Wójcik Dawid

**Affiliations:** 1 Institute of Agriculture, Department of Agronomy, Warsaw University of Life Sciences, 159 Nowoursynowska Str, 02-776 Warsaw, Poland; 2 Institute of Agriculture, Department of Biometry, Warsaw University of Life Sciences, 159 Nowoursynowska Str, 02-776 Warsaw, Poland; 3 Biebrza National Park, Osowiec-Twierdza 8, 19-110, Goniądz, Poland

**Keywords:** Fen meadow, Floods, Grazing, Land-use change, Mid-forest grasslands, Rewilding

## Abstract

Free-ranging grazers are increasingly being introduced to areas of high natural value, such as wetlands. There is also growing attention that has been paid to the historical role of herbivores in shaping ecosystems and landscapes. Even though studies on the grazing of free-range horses were carried out in different regions and climates, still little is known about their habitat selection on heterogeneous marshy areas in the temperate region of Europe. The aim of this study was to investigate the habitat use by contemporary Konik horses during the growing season on the basis of three-year GPS data for a semi-feral herd kept on wetlands. Almost 68% of the occurrence of Konik horses were in open habitats. The assessment of habitat selection by the horses confirmed their strong preferences for grasslands on mid-forest dunes and forest avoidance. Konik horses somewhat preferred mowed fen meadows, but the animals displayed differences in the selection of habitat, probably depending on its humidity and weather conditions in a given year which may limit the role of grazing in protecting these communities. Horses need different habitats in wetlands such as fen meadows, forest, and grasslands on the mineral hills. This should be taken into consideration for landscape management in areas where the introduction of wild or semi-wild horse populations is planned.

**Supplementary Information:**

The online version contains supplementary material available at 10.1007/s10661-023-11605-y.

## Introduction

In recent years, free-ranging grazers are increasingly being introduced to areas with high natural value in Europe (e.g., Naundrup & Svenning, 2015; Doboszewski et al., 2017; Reķe et al., 2019a) where the progressive loss of traditional agricultural landscape enhances the rewilding of the area (Navarro & Pereira, 2012). Furthermore, there is a growing attention that has been paid to the historical role of herbivores in shaping ecosystems and landscape, which translates into means toward the reintroduction of selected species (Reķe et al., 2019b). Cattle and horses are of particular importance in this thanks to their adaptability to a wide variety of climatic conditions (from cold boreal to warm tropical). These species can also play the role of aurochs *Bos primigenius* and wild horses *Equus ferus—*extinct grazers which formerly inhabited respective forests (Vera et al., 2006, Lovász et al., 2021). Rewilding following the introduction of free-range grazing in Europe can help to preserve habitats and maintain or restore open landscape. This process particularly concerns marginal areas in the temperate climate zone, preservation of which requires continuation of previous extensive management (Joyce, 2014). Such habitats include wetlands, which play a special role among semi-natural grassland ecosystems in the landscape of Europe not only as the carbon fixation storage or for nutrient and contamination retention but, due to their importance, for biodiversity conservation (Verhoeven et al., 2014). Extensive grazing can contribute to slowing down of the reed or willow-birch thicket encroachment and lead to an increase in biodiversity (Sienkiewicz-Paderewska et al., 2020). On the other hand, there is a risk of lowering the richness of species as a result of foraging and trampling (e.g., Stammel et al., 2003).

When comparing horses and cattle, the first species seems to be more suited to grazing on wetlands. They are characterized by their lower weight, thus lessening the risk of soil compacting (Cromsigt et al., 2018), and have the better ability to compensate for lower nutrient content through higher fodder intakes and lower methane emission than livestock (Duncan et al., 1992; Rook et al., 2004). In almost all rewilding areas in Europe, the climate and available habitats are suitable for horses and should allow for the maintenance of their viable populations (Naundrup & Svenning, 2015). Depending on the region, different breeds are suitable for introduction. Among them, the local ones — such as the Exmoor pony in Northwest Europe and England, Konik polski (Polish primitive horse; Konik) in Central and East Europe, Hucul in the Carpathians, or Pottoko in the mountains of Southwestern Europe — play an important role because they are well adapted and still used to living in the wild (Linnartz & Messner, 2014). The maintenance of horses in the natural environment is served, among other things, by establishing the refuges in which animals live in a large enclosure. In such reserves, their contact with humans is limited to periodic winter feeding, if necessary, and exclusion of the offspring in order to prevent inbreeding (Górecka-Bruzda et al., 2020).

The need to use grazing as a form of natural environment protection became a reason for the establishment of a Polish primitive horse refuge in the Biebrza National Park (BbNP) situated in northeastern Poland. It is the largest national park in the country, protecting the Biebrza river valley with a total area of c. 60,000 ha covered also by the Natura 2000 network site Ostoja Biebrzańska (PLB200006) and by the Ramsar Convention. Many habitats of the Biebrza valley were shaped by the traditional, extensive use for haymaking which took place from the 16^th^ century until the second half of the 20^th^ century. During the last 50 years, the use of Biebrza meadows has been gradually abandoned due to the difficulties in conducting agricultural management, its low profitability, and depopulation of the countryside. The cessation of the mowing and grazing of grassland communities led to their encroachment by common reed (*Phragmites australis*) and woody vegetation, including willows (*Salix* sp.) and birches (*Betula* sp.; Berezowski et al., 2018). Therefore, the secondary succession on wet and marshy meadows is one of the main threats to the biodiversity of BbNP.

When establishing new horse refuges, it is important to monitor their behavior in order to maintain their well-being and to minimize the negative impact of animals on valuable natural areas. As demonstrated by various authors (e.g., Kownacki et al., 1978; Boyd, 1988; Gudmundsson & Dyrdmundsson, 1994; Crane et al., 1997; Fleurance et al., 2001; Prończuk et al., 2020), horses kept in reserves, including Koniks, spend an average of 50–70% (that is 12–17 hours) of their daily time grazing, 20–30% resting, and 10% playing. The activity and habitat use by horses depend on different factors: the time of year, time of day (night-day), the nutritional value of available communities, weather conditions, the presence of annoying insects, and the distribution of watering places (e.g., Duncan, 1985; King, 2002; Łojek et al., 2009; Pikuła et al., 2019). The grazing horses need diversified habitats to function properly: meadows for grazing, dunes for rest, and forest for shelter (Duncan, 1985; Łojek et al., 2009; Chodkiewicz & Stypiński, 2011a, b). Among different habitats, the non-forest communities are the main places where they stay and feed (Jezierski & Jaworski, 2008).

Even though research on the grazing of different wild-roaming or free-range horse breeds was carried out in various regions of the world, in different climates (e.g., Crane et al., 1997; Linklater et al., 2000; Fleurance et al., 2001; Menard et al., 2002; Hampson et al., 2010a, b; Shingu et al., 2010; Collins et al., 2014; Kaweck et al., 2018), still little is known about the scope for grazing of feral horses on heterogenous wetlands in the temperate region of Europe an example of which is the refuge in BbNP. Foregoing research projects were based on the direct visual observation of grazing animals and focused on their behavior and habitat selection in a rather short-term time scale (Auer et al., 2021). Currently, the Global Location System (GLS) is increasingly used to monitor the distance traveled by animals, to assess the size of their territory, and their spatial utilization of habitats. Surveys with the use of collars with a GPS receiver have been carried out on wild-roaming horses (e.g., Kaczensky et al., 2007; Hampson et al., 2010a, b), but most of them were conducted under short-term projects (from 6.5 days to several weeks). Telemetric methods were used only a few times in one-year or longer studies in Europe (Berger et al., 1999; Kerekes et al., 2019; Lagos & Fagúndez, 2022) on the activity of horses.

The aim of this study was to describe the habitat using patterns of contemporary Konik horses during the growing season on the basis of three-year GPS data for a semi-feral herd kept on wetlands in Biebrza river valley. The main research questions are as follows. How do semi-feral horses (moving freely during the vegetative season from May to September) use the area of large, heterogeneous wetlands? Are there seasonal patterns of spatial use of available habitats by horses? What effect do the temperature and spring flooding have on the frequency of locating horses in particular habitats?

## Materials and methods

### Study site and climate conditions

The BbNP protects a vast peat depression of the Biebrza river valley with a total area of c.60,000 ha dominated by the wetland communities. The park is divided into three basins: Upper, Middle, and Lower. We conducted the study in years 2019–2021 in the Grzędy forest district (53°61′88 N, 22°76′54 E) which covers about 4839 ha in the Middle Basin of BbNP. The park area is not fenced.

**Fig. 1:**
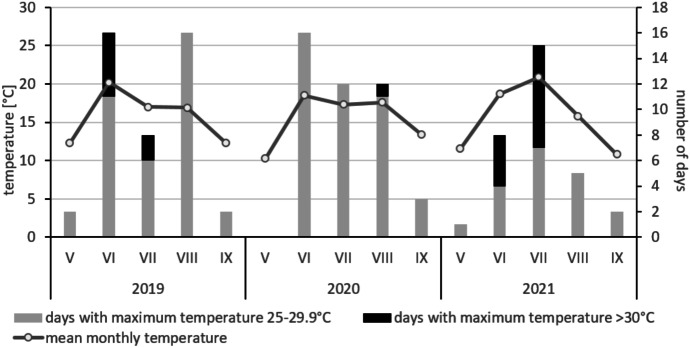
Average monthly temperature since May till September in years 2019–2021 (data from weather station Biebrza, Poland) (IMGW Data, 2019; 2020; 2021)

The climate in the Biebrza valley is temperate continental with the mean annual temperature and rainfall of 6.6^○^C and 583 mm, respectively (Wiśniewski et al., 2001). The extreme instantaneous air temperatures range from −25 °C in winter (January–February) up to over 30 °C in summer (July–August; Grygoruk et al., 2014). The growing season lasts for ca 205 days (Górniak, 2004). Our study was conducted from May to September in three years 2019–2021. Although the average temperatures during this period (V-IX) were similar for all years, they were slightly different as regards the average temperature in particular months (Fig. 1). The first year of the study was characterized by warm May and June (with the highest number of hot days and highest average temperature — 20.2 °C— in the latest month, Fig. 1). In 2020, during all summer months, the average temperatures ranged between 17.3 and 18.5 °C. July of 2021 was extremely hot, with the average temperature over 3 °C higher than in previous two years. It resulted from the highest number of the days with the temperature over 30 °C (Fig. 1). In contrast, August and September in 2021 were much colder—even on average 1.5–2° colder than in 2019 and 2020.

**Fig. 2:**
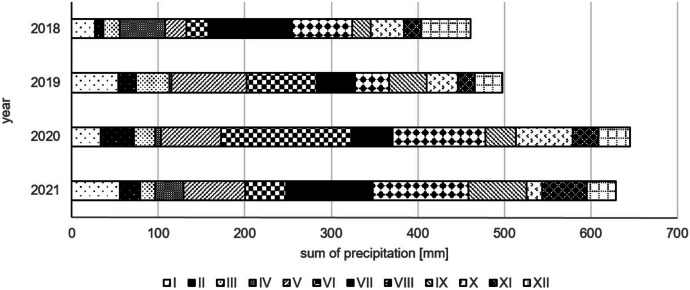
The total monthly precipitation in the years 2018–2021 (data from a weather station on Biebrza, Poland) (IMGW Data, 2018; 2019; 2020; 2021)

**Fig. 3:**
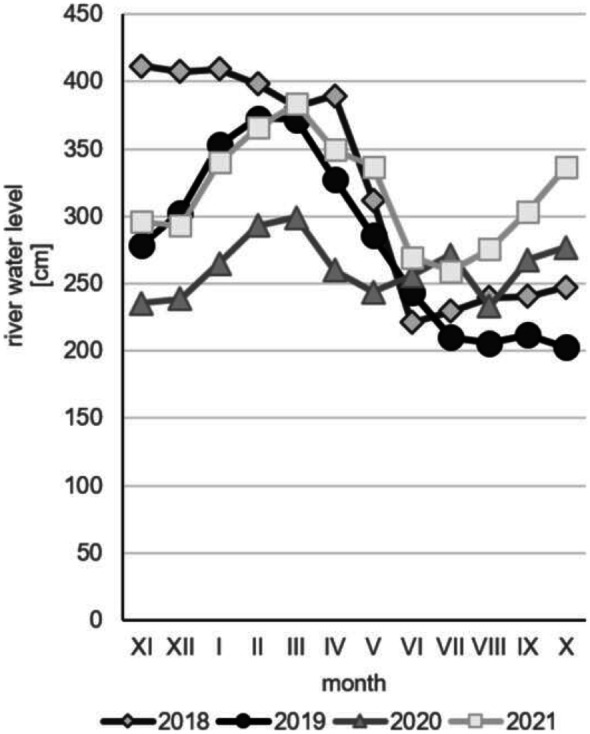
The Biebrza river water level in the years 2018–2021 (data for hydrological years from the Osowiec station, Poland) (IMGW Data, 2018; 2019; 2020; 2021)

The particular years also differ regarding water conditions. Meadows situated in the Middle Basin of the Biebrza Valley are usually flooded in springtime and water stays on the ground from March to May or early June. The duration of flooding is related to the water level in the river and the amount of precipitation. In spring 2021, there was a severe drought and the fen meadows were not flooded. This was a consequence of the small amount of rainfall in the previous years (Fig. 2) and was related to the very low Biebrza river water level (Fig. 3). On the other hand, 2021 was characterized by a high water level (Fig. 3). The floods lasted until the beginning of July and were accompanied by an extreme amount of harassing insects (*Haematopota* sp., mosquitoes, and others; own observation).

### The Konik horse refuge in BbNP

The Centre of Conservation Breeding of Konik Polski Horses and Rehabilitation of Wildlife in BbNP was established in 2004 in the Grzędy forest district. Konik horses belong to a group of breeds that are resistant to adverse environmental conditions, able to use low-quality forage, and capable of compensating for periodic food shortages. During the first 13 years, the horses were kept in a 210 ha enclosed rangeland area. Since 2017, the fence has been removed and the herd of Koniks has been moving freely during the vegetation season within the territory of the Grzędy forest district. Koniks are well adapted to the BbNP conditions. The animals stay in a single herd, where the direction of migration is decided by mares — mainly the alpha (Jaworski & Golonka, 2003). The stallion guards and defends the herd (the harem of mares with foals) from danger and plays the role of a sire. In the years 2019–2021, the herd consisted of 10 adult mares, one stallion, and their offspring. According to the rules of reserve breeding of horses (PHBA-Polish Horse Breeders Association, 2020), the animals have been kept with as little human intervention as possible, limited to herd population control or hay provision in the food shortage period. Thus, Konik horses in BbNP are semi-feral ones because during the growing season they live in a feral state, whereas during late autumn and winter (time of supplementary feeding with hay) they have contact and experience with humans (but still they are kept outside all year round). It is worth noting that the horses coexist in BbNP with other large herbivores: elks, red deer, and roe deer. Predators represented by wolves and lynxes also occur in the Central Basin of the Biebrza river (own observation). The large share of marshland habitats and the horses being allowed to roam freely within the area make the breeding system unique to Poland and Europe.

### Habitat types

**Fig. 4:**
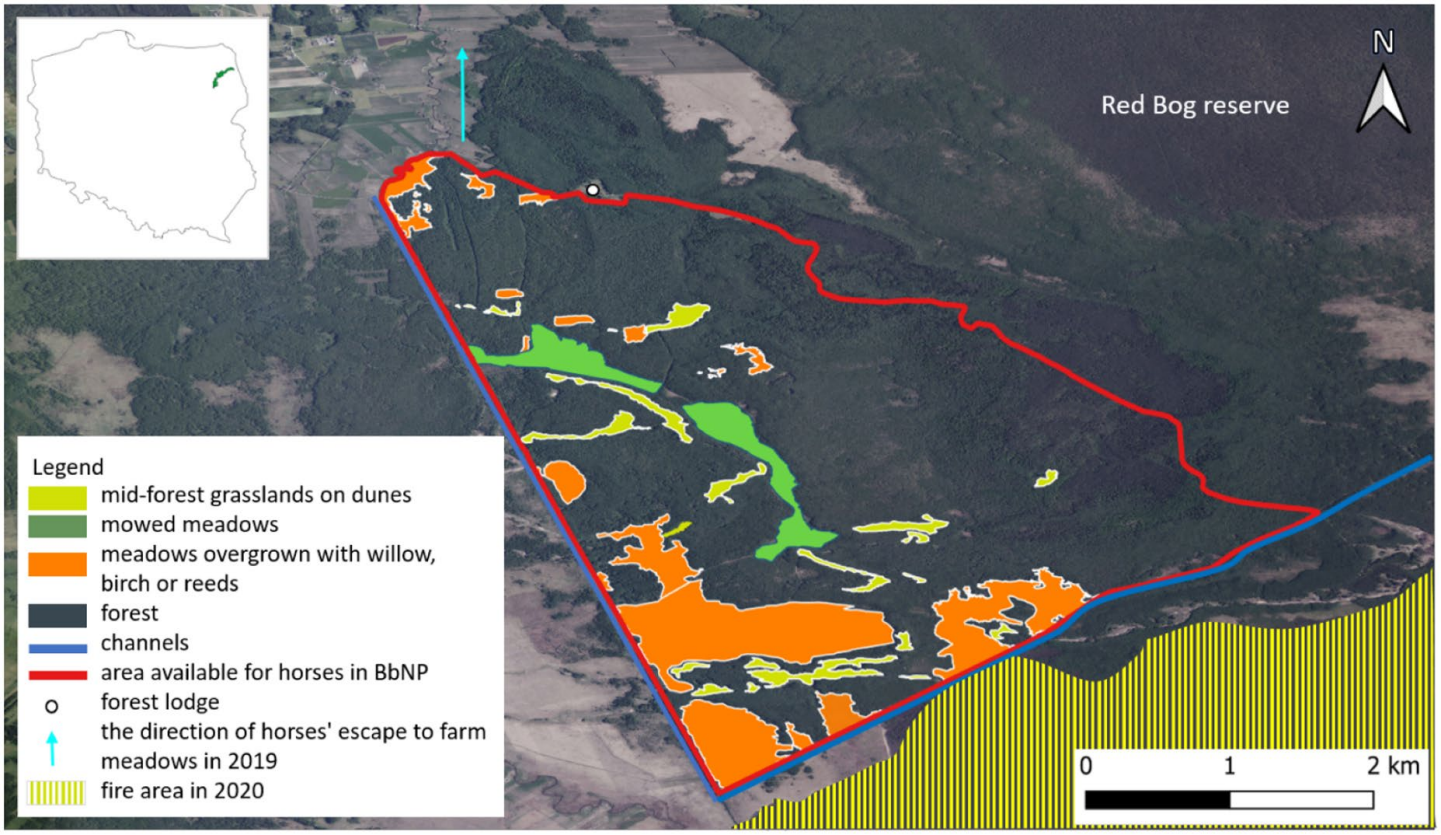
The localization of the Biebrza National Park in Poland (upper left corner) and the habitats within the area potentially available for Konik horses in the BbNP

We estimated that the area available for Konik horses in the Middle Basin of BbNP covers about 2248 ha. Although it is not fenced, it is possible to determine its natural boundaries. These are the Woźnawiejski Canal (Kanał Woźnawiejski) to the west, a small canal (Rów spod Polkowa) to the south, and the Red Bog forest reserve (Czerwone Bagno) — the oldest low peatland reserve in Poland— to the east and northeast (Fig. 4). There are four types of habitats here (Table 1): mid-forest grasslands on dunes, mowed meadows, overgrown meadows, and forest.

**Table 1:** Habitats available for horses in the Middle Basin of BbNP

Habitat type	Area	
	ha	%
Mid-forest grasslands on dunes	61	2.7
Mowed meadows	75	3.3
Overgrown meadows	298	13.3
Forest	1815	80.7
Total	2248	100

The Middle Basin of BbNP where Koniks are freely walking during the vegetative season is a large forested area (Fig. 4), dominated by mixed bog forests and alder forest with an admixture of wet forest and coniferous bog forest (Forest Data Bank, 2023). On the mineral hills — the drier areas — fresh coniferous forest and fresh forest are present. The forest stands consist mainly of downy birch (*Betula pubescens*) and black alder (*Alnus glutinosa*), whereas in the fresh habitats silver birch (*Betula pendula*) and common ash (*Fraxinus excelsior*) have a greater share (Forest Data Bank, 2023). Among the non-forest habitat types (Fig. 4), there are different fen meadows. The two big complexes (north and south) with an area of approx. 75 ha with sedge communities of the *Scheuchzerio-caricetea nigrae* and *Molinio-Arrhenatheretea* classes prevailing (Sienkiewicz-Paderewska et al., 2020; Chodkiewicz et al., 2023) are regularly mowed once a year and usually flooded in spring. They are situated on typical slightly acidic peatland soil with high soil organic matter content (Sienkiewicz-Paderewska et al., 2020). Another 298 ha are semi-open, former meadows that are being overgrown with willow (*Salix* sp.), birch (*Betula* sp.), and/or reeds (*Phragmites australis*). These areas in different succession stages are situated mainly in the southern part of the area and near the Woźnawiejski Canal of the Middle Basin of BbNP. Moreover, there are 20 mid-forest grasslands of different sizes with a total area of approx. 60 ha located on dunes. The dunes are hillocks — mineral-soil islands — elevated from the wetlands and forming habitat of intermediate humidity at edges and dry at the top. At the top of most elevations, there are patches of *Corynephorus* communities (Natura 2000 habitat 2330) with different degrees of development, while the slopes are covered by a mosaic of patches with a prevalence of bushgrass (*Calamagrostis epigejos*), acute sedge (*Carex acuta*), or grasses: smooth meadow grass (*Poa pratensis*) or downy oat-grass (*Helictotrichon pubescens*) (own data, unpublished). For bird protection reasons, the employees of the park mow the meadows and grasslands on dunes once a year, in late August/early September. All measurements of the area of grasslands on dunes and meadows were done using the QGIS 3.18.2 software.

### Data collection and statistical analysis

During the study, one Konik horse kept in BbNP wore a collar with a GPS receiver (ECOTONE) which provided information on their location at six-hourly intervals (four times a day, i.e., at 00:00 a.m., 06:00 a.m., 12:00 p.m., and 6:00 p.m. hours). Due to the fact that horses stayed in one group, the data from one receiver, worn by a randomly chosen mare, represented the location of the whole herd. Periodically, the data were confirmed by monitoring the occurrence of horses in the field (e.g., direct observation, left dung). The data collected covers the period from May to September in the years 2019–2021. In 2019 and 2020, Konik horses in the early winter left the BbNP area (Fig. 4) and grazed on the meadows belonging to a local farmer and interventions of the park employees were needed. Due to repeated escapes, the animals spent part of the winter in an enclosure near the forester’s lodge. In spring 2020, due to the drought, there was a massive fire in the Middle Basin of Biebrza river valley (Fig. 4), and because of this, the horses were kept for safety reasons in an enclosure near the forest lodge and released at the end of April.

All location points were assigned to habitat (concrete dunes, meadows mowed or overgrown with willow-birch thicket and reeds, forest) using the QGIS 3.18.2 software based on the orthophoto map for Poland (Geoportal, 2021). The area used by Konik horses was determined by measuring the distance between the most distant locations in the north, south, east, and west of the region. The GPS receiver was equipped with a thermometer which recorded the temperature at the same time as the location data and was connected with the thermal condition experienced by horses (e.g., the temperature recorded was higher when the horse stayed on the dune in the full sun and lower when it hid in the forest). In order to determine how the temperature influenced habitat preferences of Konik horses, the temperature ranges were defined as <10 °C — very cold, 10.0–14.9 °C — cold, 15.0–19.9 °C — warm, 20.0–24.9 °C — very warm, and ≥30 °C — hot.

Correspondence analysis (CA) was used to evaluate the differences in the frequency of Koniks’ location in individual regions (meadows, east, south, north, and middle) or habitats (forest, meadows, dunes, willow-birch thickets) during the year, in individual months, based on the temperature and daylight (fixes from 6 a.m., 12 p.m., and 6 p.m. in contrast to the night represented by 12 a.m.). The corresponding analysis was carried out based on the frequency table and results were shown on biplots. For this analysis, we used the FactoMineR and factoextra packages in the R 4.2.1 software. It also assessed the relative preference for and avoidance of the four habitats by Konik horses: dunes, meadows, forest, and thickets. The preferences were calculated according to Jacobs’ index of selection (JI, Jacobs, 1974) defined as

$$\textrm{JI}=\left({o}_i-{p}_i\right)/\left({o}_i+{p}_i\right)$$

where *o*_*i*_ is the proportion of use of the habitat (% of locations in a given habitat) and *p*_*i*_ its proportion of availability. We calculated the *p*_*i*_ for particular habitats as their percentage share in the whole area potentially available for horses (shown in Fig. 4). We calculated the area of each habitat with the help of the QGIS 3.18.2 software based on the orthophoto map for Poland (Geoportal, 2021). JI ranges between +1 which means maximum preference and −1 for maximum avoidance.

## Results

**Fig. 5:**
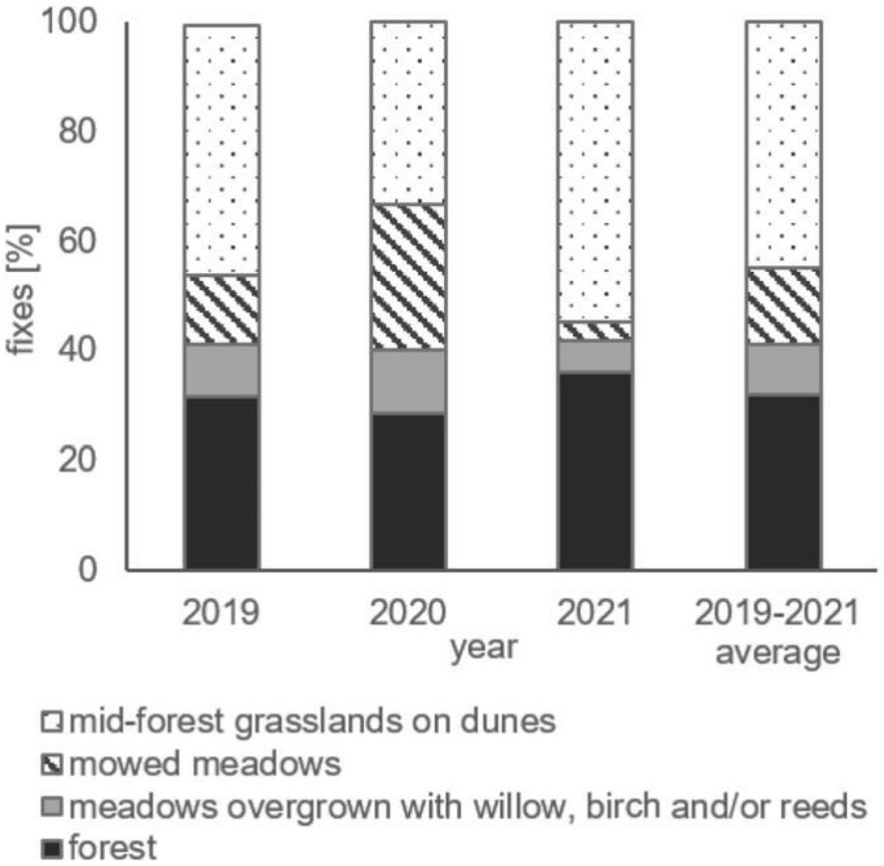
The percent of fixes (horses’ location) on the different habitats from May to September

The data that we collected contained 1466 GPS positions in total from 448 days (out of 459 possible in the three years from 1^st^ May until 30^th^ September). Thus, the fixes acquisition was 80% (over three years, on average 3.2 successful fixes per day). From May to September, horses remained mainly in the open habitats (in general, 67.8% of fixes; Fig. 5) whereas for the rest of the time in the forest. Animals were most often located in mid-forest grasslands situated on dunes (44.7% of fixes), of different sizes and with different tree stock density. Out of 20 grasslands on dunes available, Konik horses used as many as 17 with the total area of approximately 57 ha. The second habitat in which the animals were frequently located was the forest. However, we noted that Konik horses stayed usually under the tree canopy but close to the mid-forest grasslands or meadows. Considering the point occurrence, there were no fixes which located the animals in the dense forest between the mowed meadows and the Red Bog reserve. Konik horses also more often stayed in the mowed meadows than in those overgrown with willow-birch thickets and/or reeds.

**Fig. 6:**
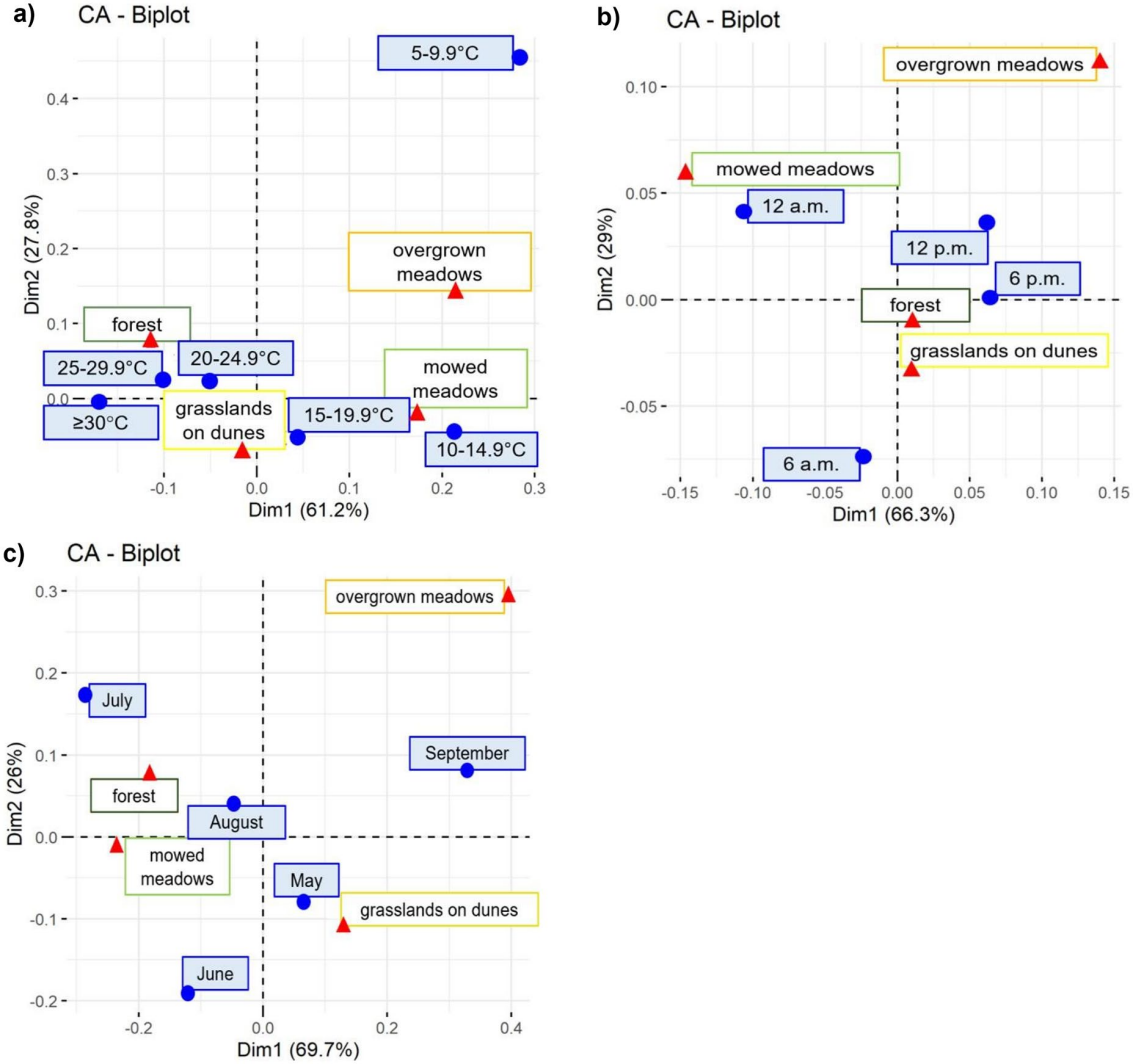
Frequency of Konik horses being located in different habitats depending on temperature (**a**), time of day (**b**), and month (**c**). The distance between any horses’ location points or habitat-depending points gives a measure of their similarity (the smaller the distance, the more similar) or dissimilarity (long distances)

The frequency with which horses stayed in different habitats was related to temperature. When the temperatures exceeded 20 °C, horses looked for shelter in mid-forest grasslands on dunes and in the forest (Fig. 6a), whereas cooler temperatures meant that the animals remained in the sedge meadows. This is also indicated by the more frequent location of horses in this latter habitat at midnight — when it is generally cooler — than in the daytime (Fig. 6b).

**Fig. 7:**
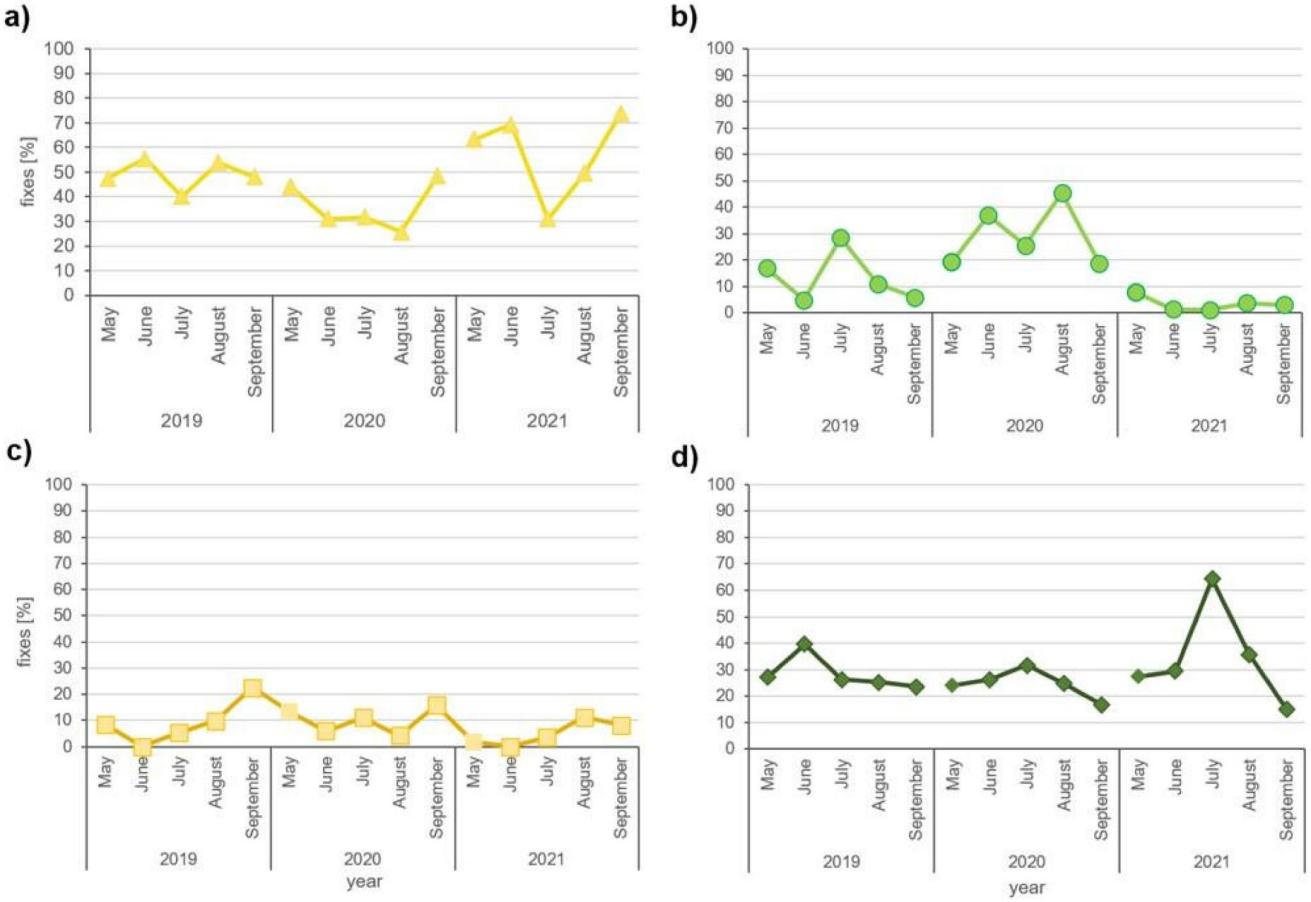
The percent of horses’ location in **a** dunes, **b** mowed meadows, **c** meadows overgrown with willow, birch and/or reeds, and **d** forest in V-IX in years 2019–2021

On average, during the growing season, the horses were more often located on dunes in May, June, and September than in the remaining summer months (Fig. 6c). On the other hand, there were relatively more fixes in the mowed meadows and in the forest in June, July, and August in comparison with spring and early autumn. This pattern has not been observed over all three years (Fig. 7). The temperature and probably the occurrence of flooding had an impact on the presence of animals in respective habitats (Fig. 7). Horses were more often located in meadows in 2020 — the dry year with a small number of hot days and with a maximum temperature exceeding 30 °C. In contrast, in 2021 — characterized by the high water level and very hot July — they were more likely to stay in the dunes and in the forest. This is indicated in particular by the frequency with which the horses were located in these habitats in the months with the highest number of days with the maximum temperature exceeding 25 °C — that is in June 2019 and July 2021 (Fig. 7).

**Fig. 8:**
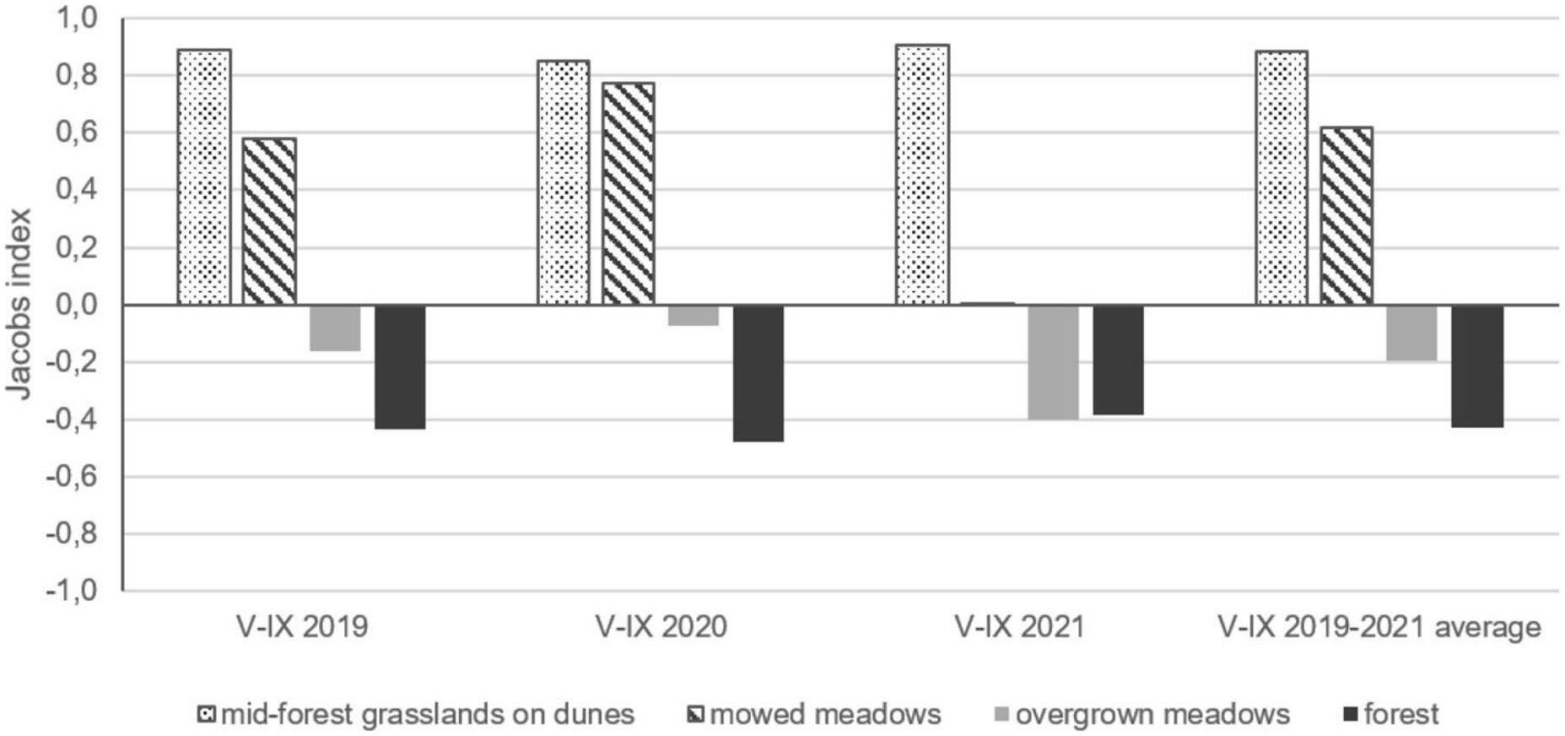
Habitat preferences of the Konik horses in BbNP in the period from May to September in the years 2019–2021. The Jacobs’ index was calculated as the ratio of the percent locations in the given habitat in a particular growing season and as a three-year average to the share of this habitat in the available area for horses in the Middle Basin of Biebrza river

The calculated values of the Jacobs’ index indicate that semi-feral horses on wetlands select mainly dry, mid-forest grasslands situated on dunes, whereas the preference toward mowed fen meadows depends on the year (Fig. 8). In the dry year—2020—mowed meadows were almost as preferred as mid-forest grasslands (Jacobs’ index value near 0.8). In contrast, in 2021, horses were located in the fen meadows almost proportionally to the share of this habitat in the area used. The meadows overgrown at different successional stages were avoided by the animals but, similarly to the mowed meadows, it might depend on the humidity conditions in a given year.

**Fig. 9:**
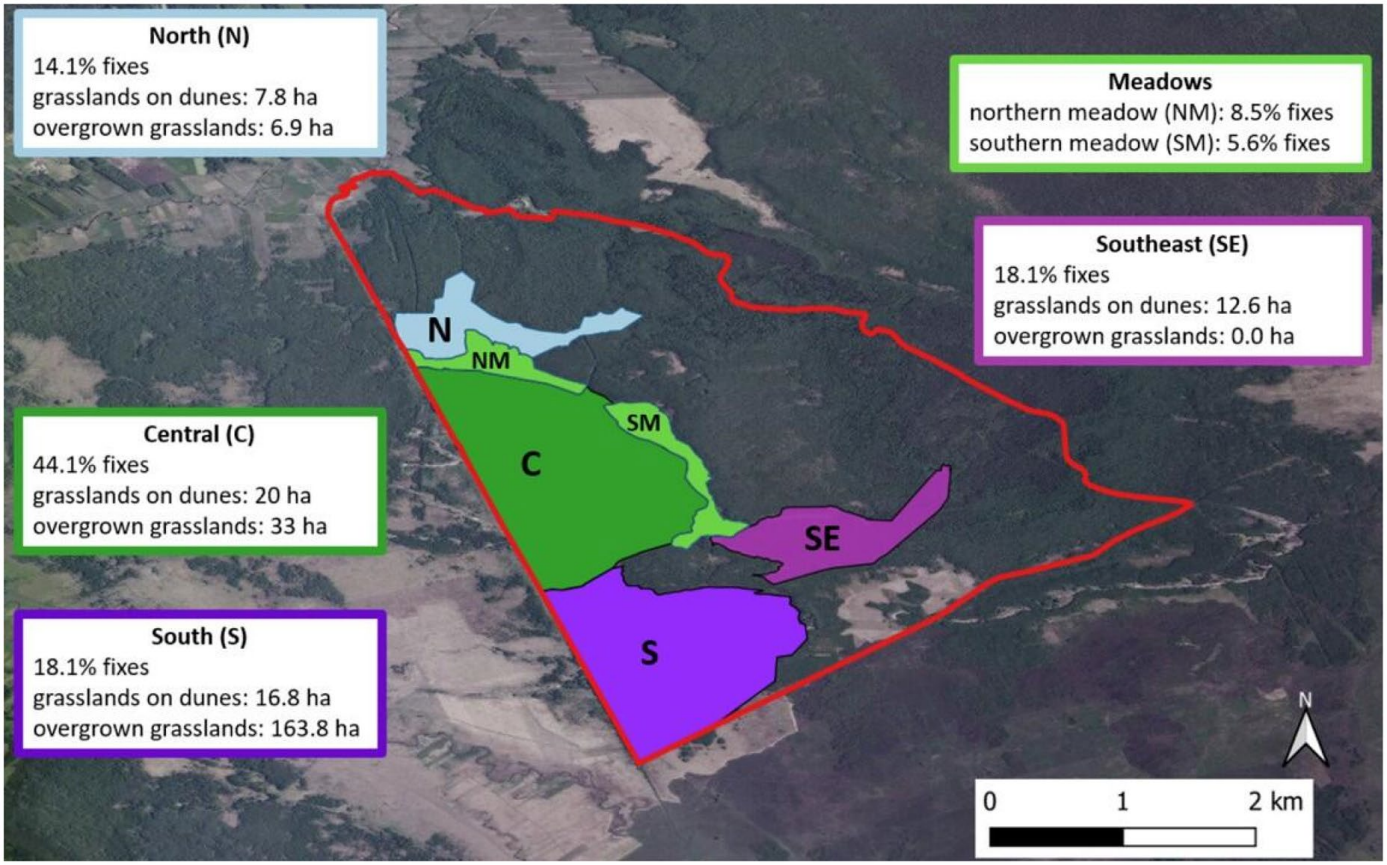
Frequency of locating horses and area of open habitats in distinguished regions

**Fig. 10:**
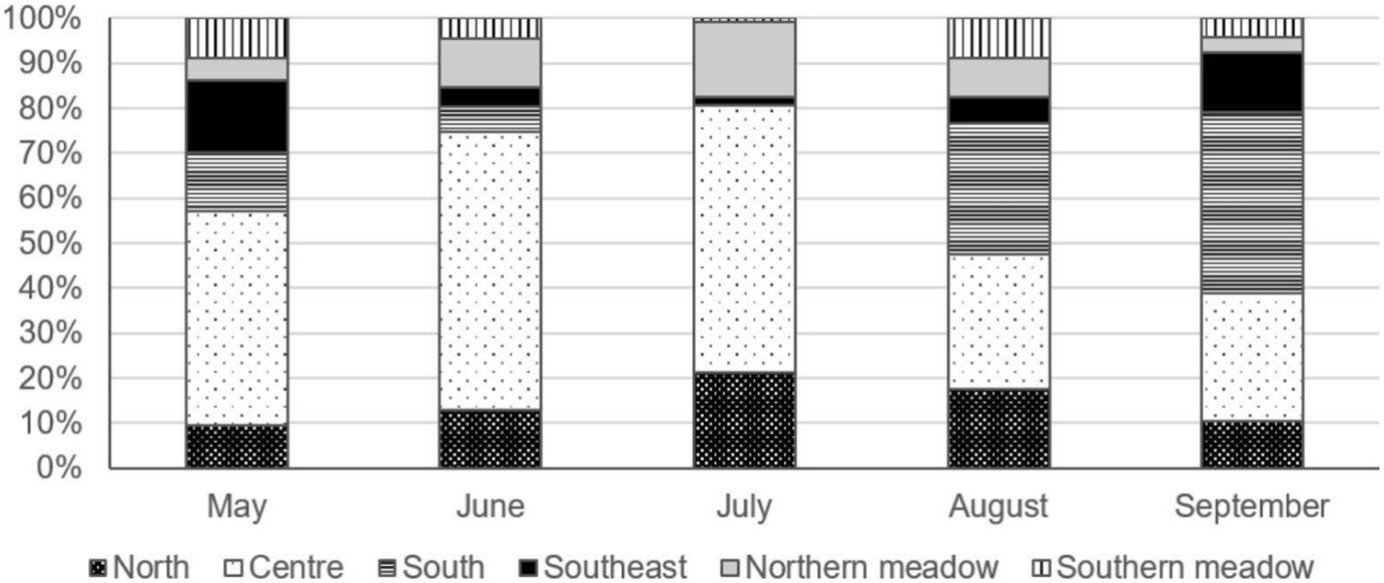
Frequency of location of the horses in individual regions over the growing season (2019–2021 average)

Data for the years 2019–2021 show that Konik horses use an area of approx. 6.15 km along the north–south axis and 3.60 km east–west. The free-roaming Konik horses during the growing season use the available area unevenly. Considering the landform, the number, and the area of mid-forest grasslands on dunes, five regions (subareas) used by Konik horses may be distinguished: north, central, south, southeast, and meadows (Fig. 9). The estimated total area of all these regions is 800 ha, which can be considered as the area used by horses in the BbNP during the vegetative season. During the vegetative season, the animals stayed mainly between the Woźnawiejski Canal and mowed sedge meadows, where a mosaic of forest and dunes prevails (the central region). Horses were located there mainly from May to July (respectively, from 47% to about 60% of fixes on average) whereas in the second half of the growing season the points of occurrence were half the size (Fig. 10). This region was characterized by the highest area of the grasslands situated on dunes (6 mid-forest grasslands with a total area of 20 ha).

The animals stayed less frequently in other regions. The frequency of location of the horses in the northern region has been increasing since May, peaking in July with the points of occurrence twice higher than in springtime (Fig. 10). It corresponds to the frequency of animals staying in the northern meadow which was used by them mainly in June and July. In general, the frequency of location of the horses in meadows increases as the growing season progresses — from 14% (in May) to 18% (in August). However, in contrast to the northern meadow, the horses were most often localized in the southern sedges in May and August. The animals were less likely to stay in this habitat in September (on average only 8% of fixes), which probably results from the fact the meadows are mowed in the first decade of this month. The beginning (May) and the second half of the growing season (August–September) are the time when horses were more often located in the southern part of their area. In particular, this concerned the southern region, characterized by 5 mid-forest grasslands on dunes. Their area is almost 17 ha (slightly less than in the central region), but they are distant from the meadows in a straight line by as much as about 1 kilometer. In all three years, horses migrated to the south and stayed there for several dozen consecutive days. In 2019 and 2020, this took place at the very beginning of September, which corresponded with the date when the mowing of the fen meadows started. In 2021 — with an exceptionally cold August — the migration of horses took place earlier, on 16^th^ August.

## Discussion

67.8% of fixes collected during three consecutive growing seasons in BbNP were in non-forest habitats, which is also consistent with previous observations of the animals in other refuges (e.g., Jezierski & Jaworski, 2008) and emphasizes their importance for the horses. During the vegetative season, Konik horses stayed mainly on mid-forest dunes. The use of as many as 17 such grasslands out of 20 available to the animals indicates how important it is to maintain a diverse landscape when planning grazing, especially in protected areas. The mid-forest elevation as well as forest is considered as places where horses mainly rest (Duncan & Cowtan, 1980; Keiper & Berger, 1982; Łojek et al., 2009). Previous observation of animals kept on wetlands showed that such refuges are characterized by higher wind velocities and lower temperatures which contribute to lower pest densities than in the surrounding habitats (Keiper & Berger, 1982). The use of mid-forest grassland as resting areas is highly seasonal, correlated with the presence of insects and almost entirely limited to the daytime (Duncan & Cowtan, 1980). The direct visual observation of Konik horses showed that in temperatures above 25 °C the grazing time is reduced in favor of resting on dunes (Łojek et al., 2009). On very hot days, especially during periods of high insect harassment, they can take refuge not on mid-forest grasslands but in the deep woods (Górecka & Jezierski, 2007). According to our results, when the temperature already exceeds 20 °C, the animals stayed more often on mid-forest grasslands than on meadows. The increased number of horses’ location in the woods in the two warmest months, which were June 2019 and July 2021, supports the observation of Górecka and Jezierski (2007). Possibly also the presence of annoying insects in 2021 might have forced the animals to look for shelter on dunes and in the forest more often in comparison with the previous years. Moreover, the frequent stay of Konik horses on the mid-forest grasslands in BbNP, especially at the beginning (May) and end (September) of the growing season, as well as at 6 a.m. and 6 p.m. — the time when the animals usually graze the most intensively (Łojek et al., 2009) — indicates that these habitats are an important place of not only resting but also feeding. It may be supported by the fact that the prevailing plant communities on the dunes are dominated by grasses (own data, unpublished) which have rather higher nutritional values than sedges growing on fen meadows and are more preferred by Konik horses (e.g., Putman et al., 1987; Cosyns et al., 2001). In general, the presence of horses on the dunes can be considered beneficial because controlled extensive grazing is one of the recommended forms of protection of the *Corynephorus* community. By browsing and trampling on species, animals may contribute to maintaining the loose character of those communities and limit their overgrowth (Kulik et al., 2013). Considering the current number of horses, the area of individual habitats, and the frequency of animals staying in them, it should not be expected that they will contribute to the inhibition of secondary succession process in open areas. However, even now, we cannot exclude that in places where they are often present (especially in mid-forest grasslands on dunes), thanks to selective grazing, they will contribute to the increase of biodiversity, have intense local impact on flora as well as larger-scale implication for seed dispersal (Fløjgaard et al., 2021), and will slow down the process of their overgrowing.

Even though on average only 14.1% locations were in mowed meadows, they should also be considered as an important place of grazing for Konik horses. This is evidenced by the frequent stay of horses in north and central regions adjacent to this habitat during the period from May to mid-August. The animals were more often located on fen meadows at night than in daylight. In the summer months, the high temperatures and the presence of harassing insects can change the time of the horses’ activity (Duncan, 1983). It cannot be excluded that the horses compensate for the extended rest time during the day by grazing at night but this problem needs more attention and future studies.

The Jacobs’ index values indicated that both — the mid-forest grasslands and mowed meadows — are the habitats preferred by horses in BbNP. Only in 2021 — the year with the long-lasting floods — animals were indifferent in the choice of the fen meadows. Previous studies do not offer a clear answer as to the preferences of horses toward highly moist habitats and are rather based on the comparison between habitat selection in cattle (Fleurance et al., 2001; Menard et al., 2002; Loucougaray et al., 2004). Thus, according to some researchers, horses kept in European wetlands during the growing season choose communities with lower vegetation and more humid conditions than cattle do (Fleurance et al., 2001; Menard et al., 2002; Loucougaray et al., 2004). This may be due to the preference of horses for green parts of plants. In summer, when the grass communities located on elevations, exposed to solar radiation, wither, the animals spend more time in lower, wetter habitats (Menard et al., 2002). In turn, Vulink (2001), based on observations of animals grazing on Dutch polders, concluded that — unlike cattle — throughout the year Konik horses avoided grazing in very wet meadows. According to our study, it is possible that the high water level affects animals’ behavior not only directly but also has an impact on the botanical composition of the fens by favoring the growth of less palatable *Carex* species (Chodkiewicz et al., 2023). Both of these could make the fen meadow less attractive for horses.

The boundaries of the area used by Konik horses in BbNP during the growing season were similar in all years of the study; however, a certain seasonality of use of particular regions by the animals can be noticed. This is evidenced by the migration of the horses south to the dunes away from the meadows by about 1 km in early spring and autumn but not in July. During the rest of growing season, the grazing animals stayed mainly in the regions adjacent to the fen meadows. Such periodic migrations of the free-ranging horses may be dictated both by weather conditions and by the availability of the tastiest species in individual plant communities (Miller, 1983; McCort, 1984). In BbNP, it is probably due to lower temperatures at the end of the summer. Also, the deteriorating nutritional value of sedges dominant on meadows which progresses toward the end of the growing season may force them to look for communities with higher feeding values. It is rather not related to the mowing of the fens because it took place at the beginning of September, whereas horses moved to the south more often already in August, e.g., in 2019 this took place on 31^st^ August, whereas in 2021 — the year with the coldest August among studied — on 16^th^ August.

The area used by the breeding bands of wild horses depends on the availability of forage and water and may vary in size between 1 and 48 km^2^ (Naundrup & Svenning, 2015) or even up to 303 km^2^ (McCort, 1984). The herd of eleven adult Konik horses in BbNP used the area of about 800 ha (0.8 km^2^). Due to their marshy nature, the habitats in the Middle Basin of BbNP have a rather low utility value. Thus, it might prove difficult to keep a minimum viable population estimated as 72–300 individual horses (Naundrup & Svenning, 2015). In general, the minimum requirement suggested for a herd of 150 genetically diverse animals (considered as a self-sustaining population) in a fertile delta is at least 4.5 km^2^ and 45 km^2^ (Linnartz & Meissner, 2014) or even up to 3600 km^2^ (Naundrup & Svenning, 2015). Due to the fact that band sizes of wild horses usually vary between 2 and 20 individuals (Naundrup & Svenning 2015; Górecka-Bruzda et al., 2020) and their home ranges may overlap, there is still a possibility for increasing the number of horses in BbNP. It would be even recommended, taking into account the fact that the aim of the establishing of the Konik horse refuge in BbNP was to prevent or slow down the encroachment of fen meadows. The introduction of new animals should be done gradually and requires the monitoring of not only conservation status of habitats used by horses but also the conditions of the animals. The number of herds may be managed by the number of stallions (Lagos & Fagúndez, 2022). The factor that may limit the number of animals is the quality of the habitats because the two open questions still remain. (1) Are grassland communities on marshy areas sufficient to meet the feeding requirements of the horses? (2) What type of area is necessary to meet the animals’ needs, especially during long snow cover when access to plants is hindered? The results of the research on year-round grazing are varied. According to Duncan (1992), the requirements of adult horses on marshy areas were met only in April–September whereas during other parts of the year the crude protein intake was too low. In contrast, studies conducted by Gilhaous and Hölzel (2016) in Germany showed that nutritional needs of both horses and cattle can be met or even exceeded on wetlands, also in winter. The critical period for the animals is winter/early spring when in general the nutrient content in biomass is the lowest. Thus, the number of grazing horses kept in wetland refuges should be adjusted to the quantity and quality of the standing biomass in this period (Gilhaous & Hölzel, 2016). The migration of Konik horses outside the boundaries of the BbNP on the management meadows, noted in winters in 2019 and 2020, probably resulted from their search for communities with better fodder quality. It would create conflicts with farmers and problems related to financial liability for damage caused. In the case of wild game, compensation payable to farmers is regulated by the law (Hunting Law, 1995; Regulation of the Ministry of the Environment, 2019) and paid by the hunting district management or the Exchequer (in case of protected species, e.g., wisent). Horses, on the other hand, even if kept in a refuge, belong to domesticated, farm animals; thus, the responsibility rests with their owner — in this case — the national park. Feeding animals with hay limits their wanderings and causes them to stay mainly in places where the bales of hay are laid (Kownacki et al., 1978). It also could prevent the migration of Konik horses outside the park boundaries. However, supplying the animals with a large amount of hay from the meadows situated outside the BbNP should be restricted due to the risk of transporting alien plant species and providing additional source of nitrogen for fen meadows. The low stocking density of horses in the refuge should be maintained in order to prevent the negative effects of grazing resulting from excessive trampling, destruction of undergrowth, or excrement left (e.g., Stammel et al., 2003). Therefore, additionally — in accordance with the assumptions of reserve breeding — 1–2-year-old offspring mares and 1.5–3-year-old stallions should be excluded from the herd in order to prevent inbreeding (Górecka-Bruzda et al., 2020).

In BbNP, horses coexist with wolves and lynxes which potentially would regulate their population size. Even though tracks of predators were noted in the home range of Konik horses (own observations), there is no evidence of interactions between them. In northeastern Poland, domesticated animals (cows and horses) are rarely preyed on (Jędrzejewski et al., 2000). More often, wolves prey on defenseless livestock, such as sheep and cattle (Jędrzejewski et al., 2004). The diet of wolves is based on wild ungulates and depends on the availability of prey in the community. In BbNP, these predators frequently hunted red deer and moose (Jędrzejewski et al., 2012). Horses are rather unattractive because they are dangerous in self-defense, while deer are easily available (Grönemann et al., 2015). In the 70 years of history of the forest sanctuary of Konik horse in Popielno (Poland), wolf predation caused the death of a mare probably only once (Górecka-Borecka et al., 2020). It cannot be entirely ruled out that horses could be attacked by wolves, especially foals or weakened, sick horses, even though the likelihood now is rather small.

Another group of wild animals that Konik horses in BbNP coexist with are large herbivores: roe deer, red deer, and elk. Unlike them, as demonstrated by the value of the Jacobs’ index, the horses rather avoid forest even though as much as about 1/3 of all fixes collected were in this habitat. The species also do not compete with each other owing to their small populations relative to the available land area and different nutritional preferences (Klich, 2015). The diet of horses and elk can overlap only during winter (Scasta et al., 2016). In comparison with other large herbivores living in Central Europe, such as cattle and the European bison, horses strictly graze and do not need a high proportion of woody plants in their diet throughout the year (Cromsigt et al., 2018). They prefer grasses and other monocotyledonous species (e.g., Putman et al., 1987; Magnusson & Magnusson, 1990; Cosyns et al., 2001), because these contain fewer undesirable secondary compounds than herbaceous plants (Putman et al., 1987). The share of woody plants in their diet can achieve about 20% depending on the season and is the highest in winter (e.g., Klich, 2015; Cromsigt et al., 2018).

## Conclusion

Our results showed that Polish primitive horses can be successfully kept on wetlands during the whole growing season without human interference. Even though the analyses carried out refer to interval data only and do not provide information on the time the horses spend in particular regions or the way they use the habitats, it was possible to describe the impact of the temperature on the behavior of semi-feral Konik horses. The animals stayed mainly in the grasslands and fen meadows, but their preferences in a given year depend probably on the humidity of the habitat. For establishing refuges of semi-wild horses in wetland areas, it is very important that different habitats — fen meadows, forest, grasslands on the mineral hills — are available so that animals can freely choose their places of grazing and refuges depending on current weather conditions. Such frequent presence of horses on the dunes is an indication for more effective monitoring and management of horses grazing on protected areas for conserving biodiversity.

### Supplementary information

ESM 1

## Data Availability

The datasets analyzed during the current study are available in Supplementary Information file.
