# Correction to: Habitat use by semi‑feral Konik horses on wetlands—three‑year GPS study

**DOI:** 10.1007/s10661-023-12170-0

**Published:** 2023-11-28

**Authors:** Chodkiewicz Anna, Prończuk Martyna, Studnicki Marcin, Wójcik Dawid

**Affiliations:** 1 Institute of Agriculture, Department of Agronomy, Warsaw University of Life Sciences, 159 Nowoursynowska Str, 02‑776 Warsaw, Poland; 2 Institute of Agriculture, Department of Biometry, Warsaw University of Life Sciences, 159 Nowoursynowska Str, 02‑776 Warsaw, Poland; 3 Biebrza National Park, Osowiec‑Twierdza 8, 19‑110 Goniądz, Poland

**Correction to: Environ Monit Assess (2023) 195:1033**

**https://doi.org/10.1007/s10661-023-11605-y**

The original version of this article unfortunately contained an error.

**Article citation is:**

Chodkiewicz A, Prończuk P, Studnicki M, Wójcik D. Habitat use by semi-feral Konik horses on wetlands-three-year GPS study. Environ Monit Assess. 2023 Aug 11;195(9):1033. https://doi.org/10.1007/s10661-023-11605-y. PMID: 37563498; PMCID: PMC10415426.

instead of:

Anna C, Martyna P, Marcin S, Dawid W. Habitat use by semi-feral Konik horses on wetlands-three-year GPS study. Environ Monit Assess. 2023 Aug 11;195(9):1033. https://doi.org/10.1007/s10661-023-11605-y. PMID: 37563498; PMCID: PMC10415426.

